# Disseminated rhino-orbital-cerebral mucormycosis in Philadelphia chromosome-positive mixed phenotype acute leukemia: a case report and literature review

**DOI:** 10.3389/fmed.2025.1672939

**Published:** 2025-10-03

**Authors:** Yao Zhou, Meijuan He, Qiu Zhang, Jia Yao, Zheng Wang, Baoan Chen, Jiamin Guo, Fengming Gao, Zefa Liu

**Affiliations:** ^1^Department of Hematology, Xinghua People’s Hospital Affiliated to Yangzhou University, Taizhou, China; ^2^Suzhou Jsuniwell Medical Laboratory, Suzhou, China; ^3^Department of Hematology and Oncology, The Affiliated Zhongda Hospital, Medical School of Southeast University, Nanjing, China; ^4^Key Laboratory of Digital Technology in Medical Diagnostics of Zhejiang Province, Dian Diagnostics Group Co., Ltd., Hangzhou, China; ^5^Nanjing Dian Diagnostics Group Co., Ltd., Nanjing, China

**Keywords:** mucormycosis, fungal infection, rhino-orbital-cerebral, mixed phenotype acute leukemia, Philadelphia chromosome

## Abstract

Rhino-orbital-cerebral mucormycosis (ROCM) is a rapidly progressing and life-threatening fungal infection caused by fungi in the order Mucorales. It predominantly affects immunocompromised individuals, such as those undergoing chemotherapy for hematological malignancies. Despite its high mortality rate, ROCM remains underrecognized, and its clinical features in patients with Philadelphia chromosome-positive (Ph+) mixed phenotype acute leukemia (MPAL) are rarely reported. This report describes a 48-year-old female who presented with a one-week history of fever without localized pain and was diagnosed with Ph+ MPAL by laboratory blood tests and comprehensive bone marrow examination. She was treated with imatinib and received acute lymphoblastic leukemia (ALL)-like chemotherapy, and used voriconazole to prevent fungal infections. On day 9 of admission, the patient developed fever and skin lesions on the right nasal area. The skin lesions spread rapidly, indicating a potentially aggressive infection. A pathological biopsy of the affected area confirmed the diagnosis of ROCM. We administered liposomal amphotericin B (L-AmB) in a timely manner and effectively controlled the infection. The most common fungal infections in Ph+ MPAL are *Candida* and *Aspergillus*. To the best of our knowledge, this is the first case of ROCM. Our case reports support the limitations of voriconazole in preventing Mucorales infections and emphasizes the importance of broad coverage in antifungal prevention strategies, early diagnosis, and timely treatment. In addition, we reviewed 27 other cases of rhinocerebral mucormycosis in patients with acute leukemia and provide an analysis of these cases.

## 1 Introduction

Mucormycosis is a globally invasive fungal infection caused by members of the genera *Mucor*, *Rhizopus*, *Rhizomucor*, and *Lichtheimia* (formerly *Absidia*) in the order Mucorales (1). Mucormycosis rarely occurs in immunocompetent individuals but frequently occurs in immunocompromised patients (2). The incidence of mucormycosis has been increasing over the past decade, especially since the onset of COVID-19, and the condition is fatal in most patients (3, 4). Mucormycosis can present in different forms, including pulmonary, cutaneous, rhino-orbito-cerebral, gastrointestinal, and disseminated types (5). In the clinical situations, diabetes mellitus has evolved as a major risk factor for mucormycosis, while in more recent years, underlying malignancy has emerged as a critical risk factor due to the increasing number of patients undergoing chemotherapy or cancer immunotherapy (6–8).

A number of retrospective studies have shown that mucormycosis accounts for only a small proportion of breakthrough invasive fungal infection in patients with hematological malignancies (9). According to the SEIFEM study, mucormycosis is present in only 0.1% of patients with hematological malignancies (10), with pulmonary mucormycosis being the most common (11, 12). Biopsy is the preferred method for mucormycosis diagnosis but may not be an option in the early course of the disease, resulting in delayed diagnosis and missed opportunities for timely treatment (13). Antifungal therapy is usually used clinically, but it is usually difficult to treat infections effectively by drugs. Combined surgical debridement can improve the treatment effect and patient survival rate (14, 15).

Herein, we report a case of Philadelphia chromosome-positive (Ph+) mixed phenotype acute leukemia (MPAL) combined with rhino-orbital-cerebral mucormycosis (ROCM) infection, with the timely use of liposomal amphotericin B (L-AmB) to treat and control the infection. Highlighting the importance of early diagnosis and treatment of mucormycosis, it also provides important insights into the selection of prophylactic therapy for patients with hematological malignancies.

## 2 Case description

**Figure 1:**
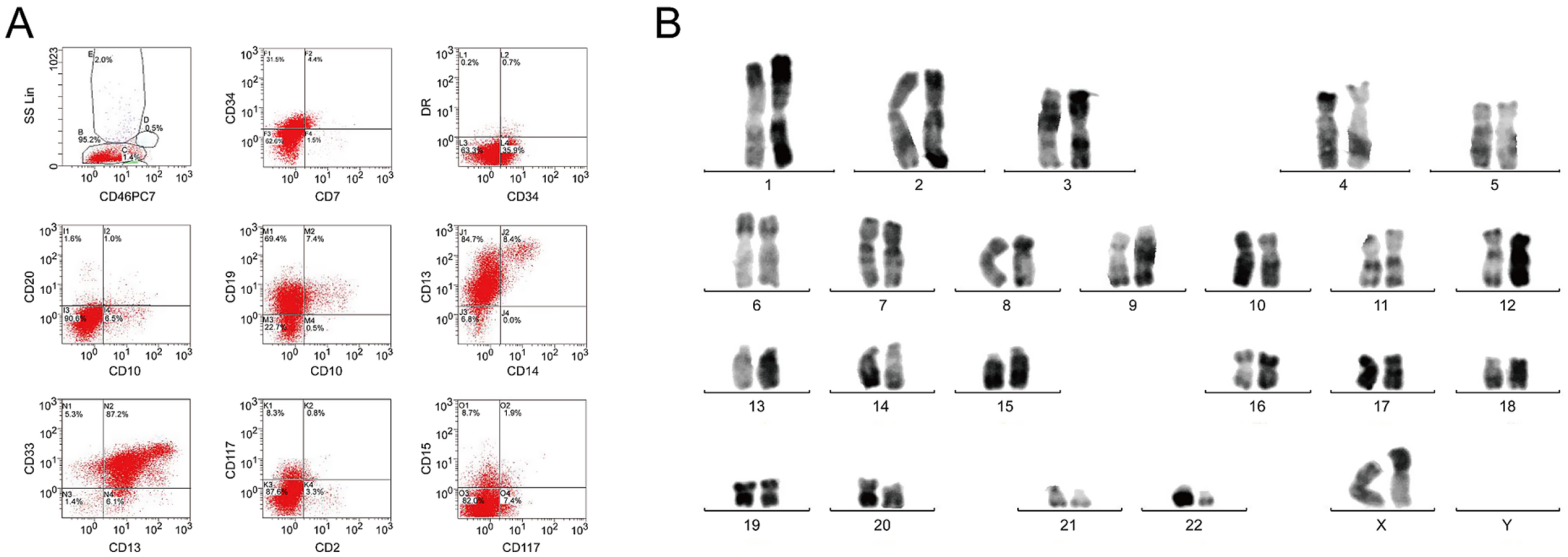
Immunophenotype and karyotype characteristics of bone marrow specimens. **(A)** Representative flow cytometry histograms demonstrate the expression of CD34, CD19, CD13, CD33, CD 11b, CD64, and CD79a. **(B)** G-banding karyotype of the BM cells demonstrates t(9;22) (q34;q11).

A 48-year-old woman who presented with fever in the absence of pain for 1 week was admitted to People’s Hospital of Xinghua City (Jiangsu Province, China). Physical examination was negative. Blood routine test demonstrated a white blood cell count (WBC) of 180.7 (4.0–10.0) × 10^9^/L; hemoglobin of 123 (110–150) g/L and platelets of 53 (100–300) × 10^9^/L. Bone marrow biopsy showed hypercellularity with 83.5% blast cells. Eosinophilia was not evident in either peripheral blood or bone marrow. Flow cytometry analysis showed that the blast cells, which accounted for 95.2% of bone marrow karyocytes, were strongly positive for CD34, CD19, CD13, CD33, CD11b, CD64, myeloperoxidase, and CD79a (Figure 1A). According to the World Health Organization (WHO) 2022 criteria, the patient was diagnosed with MPAL with co-expression of myeloid and B lymphoid lineage antigen. The chromosomal analysis of bone marrow cells revealed t(9;22) (q34;q11) translocation (Figure 1B). Molecular genetics showed BCR/ABL (e1a2) fusion gene, thereby confirming the diagnosis of Ph+ MPAL.

The timeline of diagnosis and treatment is shown in Table 1. On the 6th day of admission, the patient was treated with imatinib (400 mg/day) and acute lymphoblastic leukemia (ALL)-like chemotherapy (VDP regimen: vincristine 2 mg/qwx4; daunorubicin 30 mg/m^2^, day 1–3; prednisone 60 mg/m^2^, day 1–28). Also, preventive treatment was given with voriconazole (4 mg/kg, q12h).

**Table 1:** Timeline of events.

Time	Clinical features	Biology results	Therapy strategies
Day 1	Fever	CRP 112.6 mg/L	Hefoperazone/sulbactam 9 g/day IV and hydroxyurea 3 g/day p.o.
Day 3	Bone marrow pathology and fever	MPAL, WBC 136.8 × 10^9^/L	Cyclophos-phamide 0.4 g/day IV, dexamethasone 10 mg/day I, and voriconazole 4 mg/kg, q12h IV
Day 6	Bone marrow pathology and fever	Ph + MPAL, WBC 73.2 × 10^9^/L	Imatinib 400 mg/day, vincristine 2 mg/qwx4, daunorubicin 30 mg/m^2^ day 1–3, prednisone 60 mg/m^2^ day 1–28
Day 9	Fever again, skin lesions on the nasal	WBC 2.51 × 10^9^/L, ANC 0.1 × 10^9^/L, CRP 57.81 mg/L, CT of the chest (−)	Imipenem and cilastatin sodium 1.0 q6hIV, vancomycin 2.0 q12h, voriconazole 4 mg/kg q12h IV
Day 15	Skin lesions expand	Pathology shows mucormycosis, wound secretion culture shows Rhizomucor, (1,3)-β-D-glucan assay (−), galactomannan test (−)	L-AmB at 3 mg/kg qdIV, imipenem and cilastatin sodium 1.0 q6hIV
Day 18	Persistence of fever	WBC 0.30 × 10^9^/L, ANC 0.1 × 10^9^/L	Granulocyte-colony stimulating factor 150 μg bid subcutaneous
Day 23	Bone marrow pathology and persistence of fever	WBC 4.47 × 10^9^/L, ANC 3.5 × 10^9^/L, complete remission	Discharge home

CRP, C-reactive protein; IV, intravenous; p.o., per os; MPAL, mixed phenotype acute leukemia; WBC, white blood cell count; Ph+, Philadelphia chromosome-positive; ANC, absolute neutrophil count; CT, computed tomography; L-AmB, liposomal amphotericin B.

**Figure 2:**
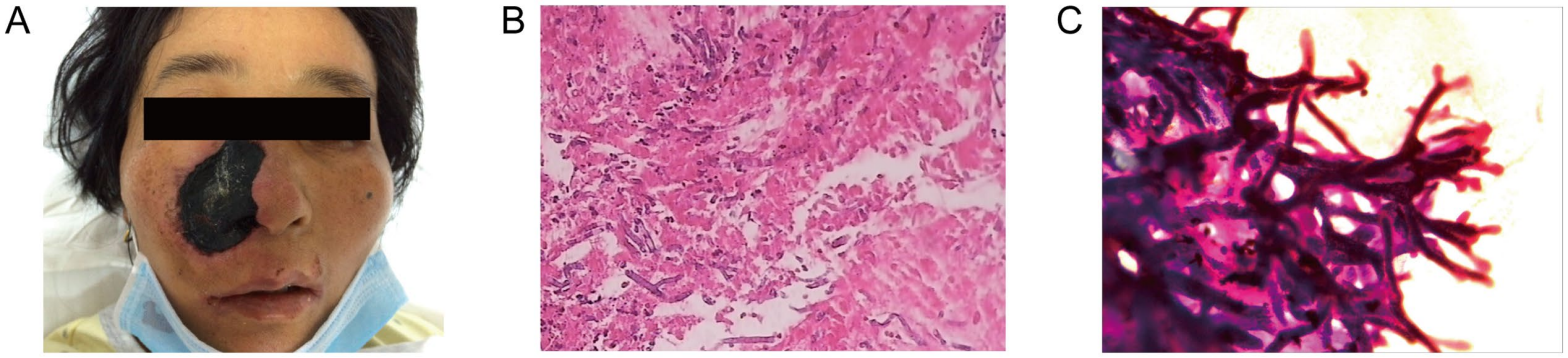
Characterization of infected tissues. **(A)** Initial clinical presentation on the whole right nasal. The skin lesion area is approximately 4 cm × 3 cm. Pathological section of necrotic tissue of right nasal cavity. Hematoxylin and eosin-stained sections show an angioinvasive growth of mucormycosis at a magnification of ×100 **(B)** and ×400 **(C)**.

On day 9 after admission, the patient’s body temperature reached 38.6 °C, and developed skin lesions on the right nasal area. The skin lesions spread rapidly to the entire right nasal area after 6 days (Figure 2A). Blood routine test demonstrated WBC 2.51 × 10^9^/L, hemoglobin 80.2 g/L, platelets 18 × 10^9^/L, and absolute neutrophil count (ANC) of 0.1 × 10^9^/L. C-reactive protein (57.81 mg/L) and procalcitonin (0.13 ng/mL) were both elevated. Chest computed tomography (CT) examination was negative, and serum (1,3)-β-D-glucan, galactomannan tests, and blood culture examinations were negative. Skin biopsy revealed hyphae consistent with mucormycosis. Wound secretion culture was positive for Rhizomucor species (Figures 2B,C). After the diagnosis of mucormycosis, according to the guidelines of the Infectious Diseases Society of America (IDSA), we administered L-AmB at 3 mg/kg/day and successfully controlled the infection. On the 23rd day of admission, the patient achieved complete remission of the infection.

We suggested bone marrow transplantation as the best option for her treatment, but she refused. The patient died of intracranial hemorrhage after 1 year.

## 3 Literature review

Because no cases of MPAL infection with mucormycosis have been reported, we searched PubMed using the terms “rhinocerebral mucormycosis,” “acute leukemia” and “case report” and found 44 articles published between 1982 and June 2025. There were 24 eligible case reports, which included complete information on a total of 27 patients (16–39). Information on age, sex, disease type, treatment, and clinical outcomes was collected for these patients (Table 2).

**Table 2:** Case data of 27 acute leukemia patients with rhinocerebral mucormycosis.

Case reports	Age and sex	Type of disease	Surgical debridement	Therapeutic drugs	Effective remission of the infection
Ding et al. (16)	46/F	AML	Yes	AmB	No
Yamamoto et al. (17)	42/M	AML	Yes	L-AmB, micafungin	Yes
Popa et al. (18)	7/F	ALL	Yes	AmB	Yes
Yang et al. (19)	1/M	ALL	No	AmB, posaconazole	No
Yeung et al. (20)	57/M	AML M5a	No	L-AmB, imipenem, amikacin	No
Samanta et al. (21)	8/M	ALL	Yes	L-AmB	Yes
Siriwardena et al. (22)	35/F	AML	Yes	L-AmB	No
Siriwardena et al. (22)	29/F	AML	Yes	L-AmB, antibiotics	Yes
Siriwardena et al. (22)	42/M	AML	Yes	L-AmB	No
Hu et al. (23)	1/M	ALL	No	L-AmB, voriconazole	No
Ojeda-Diezbarroso et al. (24)	12/F	ALL	Yes	L-AmB, posaconazole	Yes
Uraguchi et al. (25)	70/M	AML M5b	Yes	L-AmB, antibiotics	No
Wehl et al. (26)	1/M	ALL	No	L-AmB, antibiotics	No
Mutchnick et al. (27)	2/M	ALL	Yes	AmB, posaconazole, micafungin	Yes
Dworsky et al. (28)	17/F	BCP-ALL	Yes	L-AmB, micafungin	Yes
Gumral et al. (29)	40/M	ALL	Yes	L-AmB	Yes
Raj et al. (30)	55/M	APL	Yes	L-AmB	Yes
Sigera et al. (31)	19/M	B-ALL	Yes	L-AmB	No
Lerchenmüller et al. (32)	28/M	ALL	Yes	L-AmB	Yes
Cofré et al. (33)	2/F	ALL	Yes	AmB	Yes
Ammon et al. (34)	24/F	AML	No	AmB, flucytosine	Yes
Jacobs et al. (35)	35/F	AML	No	AmB	No
Jacobs et al. (35)	17/M	ALL	No	AmB, echinocandin FK463	Yes
Parkyn et al. (36)	3/M	BCP-ALL	No	L-AmB	Yes
Andreani et al. (37)	59/M	sAML	Yes	L-AmB, isavuconazole	Yes
Funada et al. (38)	21/M	BCP-ALL	No	AmB	Yes
Brusis and Rister (39)	12/M	ALL	Yes	AmB	Yes

F, female; AML, acute myeloid leukemia; AmB, amphotericin B; M, male; L-AmB, liposomal amphotericin B; ALL, acute lymphoblastic leukemia; AML M5a, acute monoblastic leukemia; AML M5b, acute monocytic leukemia; BCP-ALL, B-cell precursor acute lymphoblastic leukemia; APL, acute promyelocytic leukemia; B-ALL, B-cell acute lymphoblastic leukemia; sAML, secondary acute myeloid leukemia.

The patients, 18 males and 9 females, were mainly young and middle-aged, with a median age of 21 years (age range: 1–70 years). All patients were treated with amphotericin B (AmB) or L-AmB, and 48% were also treated with other microbicides. Surgical resection proved highly effective in controlling mucormycosis, with only 44% of non-surgical patients successfully controlled the infection, compared with 72% of surgical patients. In our case, the patients’ symptoms were effectively relieved by drug therapy alone. This encouraging result might be attributed to the timely diagnosis of Mucorales infection and the implementation of effective antifungal strategies.

This encouraging result may be attributed to the timely diagnosis at the early stage of Mucor infection.

## 4 Discussion

In 2022, the WHO classification was updated, but the main criteria for MPAL remained unchanged, except for cases defined by myeloperoxidase alone for the myeloid lineage. A new subcategory of BCR/ABL1-positive MPAL was introduced (40). In this case, the patient’s heightened susceptibility to invasive fungal infections likely arises from a confluence of immune impairments directly linked to both the disease biology and its treatment (41). Specifically relevant to this Ph+ MPAL case, the BCR-ABL1-driven tyrosine kinase activity is known to disrupt neutrophil functional capacities, including chemotaxis, oxidative burst, and phagocytosis (42, 43). Furthermore, the leukemic blasts in MPAL exhibit aberrant differentiation that disrupts normal hematopoiesis, leading to both quantitative and qualitative defects in innate immune cells (44). This intrinsic immune compromise is further exacerbated by intensive induction chemotherapy, particularly anthracycline-based regimens. Anthracyclines (such as daunorubicin) not only cause profound myelosuppression but also induce mucosal barrier injury and impair tissue-resident macrophage function, creating a permissive environment for fungal invasion (45, 46). The combination of these factors—the underlying immune dysfunction from BCR-ABL1 signaling, treatment-related immunosuppression, and mucosal barrier breakdown—creates a perfect storm for opportunistic fungal infections, necessitating robust antifungal prophylaxis strategies.

In hematological oncology regimens, voriconazole is a widely used empirical prophylactic broad-spectrum fungicide that potently inhibits Candida, Aspergillus, Scedosporium, and Fusarium, reducing the risk of invasive fungal infection in immunocompromised patients (47). However, there is evidence that voriconazole inhibition of Aspergillus creates a favourable environment for Mucorales (12, 22), associated with increased mucormycosis, and animal studies have suggested that exposure to voriconazole enhances Mucorales virulence (48). Our case provides a notable exception to the conventional management paradigm of ROCM, which emphasizes the necessity of combined surgical and medical therapy. The successful control of infection with L-AmB monotherapy, in contrast to the high surgical intervention rate observed in the literature review, warrants further analysis. We postulate that this favorable outcome is attributable not merely to the “timely adjustment” of antifungal therapy, but more fundamentally to the exceptionally early stage and superficial localization of the infection at the time of diagnosis. The patient’s initial presentation was limited to cutaneous involvement on the nasal dorsum without clinical or radiological evidence of sinusitis, osteomyelitis, or orbital/central nervous system invasion—a finding corroborated by the negative chest CT. This confined disease extent stands in stark contrast to most reported cases of ROCM in leukemic patients, which typically present with advanced sino-orbital or cerebral involvement necessitating aggressive debridement to remove necrotic, avascular tissue and reduce the fungal burden. According to the IDSA guidelines, L-AmB is strongly recommended as first-line treatment, while intravenous isavuconazole or the delayed-release posaconazole tablets are moderately recommended (49). Among them, posaconazole has emerged as the prophylactic agent of choice due to its reliable activity against Mucorales.

As observed in the current case, ROCM infection usually originates from the paranasal sinuses, with bone destruction and subsequent invasion of the orbit, eye, and brain (50). Unilateral facial edema, proptosis, and palatal or palpebral fistula developing into necrosis may be observed. When invasive fungal infection is suspected, particularly in cases of ROCM involvement, a diagnostic paradigm shift is warranted. Rather than relying on serum biomarkers such as galactomannan testing, which demonstrates poor sensitivity for mucormycosis, immediate tissue-based diagnostic approaches should be prioritized (51). Early and aggressive tissue sampling for histopathological examination, coupled with molecular diagnostics and fungal culture, is crucial for several reasons: (1) it allows definitive identification of Mucorales through characteristic histological features (broad, pauciseptate hyphae with right-angle branching); (2) enables timely differentiation from other invasive molds like Aspergillus; and (3) provides material for antifungal susceptibility testing. This diagnostic strategy is particularly critical in Ph+ MPAL patients, where delayed diagnosis of mucormycosis carries catastrophic consequences due to their profound immunosuppression and the infection’s characteristically aggressive course. This case emphasizes the need for high clinical suspicion in immunocompromised patients.

## 5 Conclusion

Mucormycosis is a rare but life-threatening infection in immunocompromised patients, particularly those with hematological malignancies. Early diagnosis and prompt treatment with L-AmB are crucial for improving outcomes. This case underscores the importance of vigilant monitoring and tailored antifungal therapy in managing mucormycosis in Ph+ MPAL patients.

## Data Availability

The original contributions presented in the study are included in the article/supplementary material, further inquiries can be directed to the corresponding author.
